# Correlating Genotyping Data of *Coxiella burnetii* with Genomic Groups

**DOI:** 10.3390/pathogens10050604

**Published:** 2021-05-14

**Authors:** Claudia M. Hemsley, Angela Essex-Lopresti, Isobel H. Norville, Richard W. Titball

**Affiliations:** 1 Department of Biosciences, College of Life and Environmental Sciences—Biosciences, University of Exeter, Exeter EX4 4QD, UK; C.Mueller@exeter.ac.uk; 2 Defence Science and Technology Laboratories, CBR Division, Porton Down, Salisbury SP4 0JQ, UK; aeelopresti@dstl.gov.uk (A.E.-L.); IHNORVILLE@dstl.gov.uk (I.H.N.)

**Keywords:** *Coxiella burnetii*, genotyping, MST, MLVA, genomic groups

## Abstract

*Coxiella burnetii* is a zoonotic pathogen that resides in wild and domesticated animals across the globe and causes a febrile illness, Q fever, in humans. Several distinct genetic lineages or genomic groups have been shown to exist, with evidence for different virulence potential of these lineages. Multispacer Sequence Typing (MST) and Multiple-Locus Variable number tandem repeat Analysis (MLVA) are being used to genotype strains. However, it is unclear how these typing schemes correlate with each other or with the classification into different genomic groups. Here, we created extensive databases for published MLVA and MST genotypes of *C. burnetii* and analysed the associated metadata, revealing associations between animal host and human disease type. We established a new classification scheme that assigns both MST and MLVA genotypes to a genomic group and which revealed additional sub-lineages in two genomic groups. Finally, we report a novel, rapid genomotyping method for assigning an isolate into a genomic group based on the Cox51 spacer sequence. We conclude that by pooling and streamlining existing datasets, associations between genotype and clinical outcome or host source were identified, which in combination with our novel genomotyping method, should enable an estimation of the disease potential of new *C. burnetii* isolates.

## 1. Introduction

*Coxiella burnetii* is an obligate intracellular, zoonotic pathogen that causes the disease Q fever in humans [1]. The bacterium can be isolated from a wide range of wild and domestic animals from almost every part of the world, including livestock such as cattle, sheep, goats, as well as domesticated animals such as cats and dogs [2]. Infection of humans occurs mainly through direct contact with infected animals or their by-products [3,4,5], although other vectors including ticks have also been suggested to transmit the disease [6]. Q fever in humans ranges in presentation from an acute, self-limiting flu-like illness, to a chronic, debilitating condition that can result in endocarditis, hepatitis and chronic fatigue [3,7]. Despite some early indications that there might be an association between genotype and acute or chronic disease [8,9,10], this has not been substantiated, and an association between animal host and disease potential in humans has not been found to date.

*C. burnetii* is considered to be a clonal species that originated from *Coxiella*-like endosymbionts of soft ticks, after acquiring the ability to infect mammalian cells [11]. Early studies showed that guinea pigs infected with isolates from different parts of the world showed different signs of disease [12,13,14,15,16]. Subsequently, Hendrix et al. [9] demonstrated that *C. burnetii* isolates collected from various sources differed in their restriction endonuclease digestion patterns of chromosomal DNA and, on the basis of these patterns, isolates could be grouped into six so-called “genomic groups” (GGs). Jäger et al. [17] later showed that these restriction fragment length polymorphism patterns corresponded to the geographical origin of the isolates, and later the term “geotype” was coined, i.e., genetic lineages and genotypes that have originated in a certain geographic location, some of which have remained locally whilst others are spreading across the globe [18]. This concept of genomic groups was later confirmed by other genotyping methods, including microarray or whole-genome sequence analyses, with four additional groups (GG VII–X) having been proposed and GG IV and GG II subsequently having been divided into further subgroups [19,20,21].

We have recently reviewed the main characteristics of each genomic group as well as their difference in virulence in animal models of disease [22]. In brief, GG I, II and III are most closely related, whilst GG V showed the highest number of single-nucleotide polymorphisms (SNPs) compared to the Nine Mile reference strain of GG I. GG IV was the most distantly related in a phylogenetic tree [21]. GG I isolates are the most virulent in animal models, followed by GG II and III, whereas isolates from GG IV and V only show moderate virulence and GG VI isolates appear to be avirulent [22]. Genomic groups also differ in their geographic distributions, with GG I and IV being found across the globe, and others being more restricted to a single continent. For example, GG II is predominantly found in Europe and GG V is found in Nova Scotia and surrounding areas of North America [18,21]. Whole-genome comparative studies have found GG-specific genome content, including differences in the repertoires of membrane proteins, T4SS effectors, and transporters, which might explain the differences in virulence and host interactions of the different lineages [21,23,24,25,26,27].

Several bacterial genotyping methods exist (see [28] for a review) for *C. burnetii*. Multispacer Sequence Typing (MST) involves PCR amplification and sequencing of intergenic, non-coding spacer sequences in the genome. Since non-coding DNA regions are more variable than coding sequences due the absence of selective pressure [29], this molecular typing method has previously been used for many other pathogens such as *Bartonella* sp., *Mycobacterium* sp., *Yersinia pestis*, and *Tropheryma whipplei* (for examples see https://ifr48.timone.univ-mrs.fr/mst/ accessed on 10 September 2020). An MST-typing scheme for *C. burnetii* was devised in 2006 by Glazunova et al. [30], and was optimised in 2011 by Hornstra et al. [10]. MST typing as a sequence-based method offers the advantage of being highly reproducible between laboratories. However, compared to other methods, it requires a higher quantity and quality of starting DNA, and is more expensive and time consuming than other methods. To date, 74 different genotypes of *C. burnetii* are included in the MST database.

Multiple-Locus Variable number tandem repeat (VNTR) Analysis (MLVA) typing involves PCR amplification of VNTR loci in coding and non-coding regions [31], and determining the repeat numbers for each locus. An MLVA scheme using seven marker loci was first described in 2006 [32]. The method was extended to 17 VNTR loci by Arricau-Bouvery et al. in the same year [33]. Different laboratories use different subsets of these loci, ranging from three to 16 VNTR regions. The MLVA6Nijmegen, which was used to study the largest outbreak of Q fever recorded in the Netherlands [34], is the most commonly used panel. A public database for MLVA genotyping data is available [35], which currently contains 379 entries. MLVA typing is more rapidly performed than MST typing since there is no need to sequence the PCR products. Additionally, multicolour, multiplex assays are available, reducing the quantity of input DNA required. However, differences in the size estimates provided by different capillary electrophoresis equipment can make comparisons between different laboratories difficult [32]. Other, less commonly used, genotyping methods exist for *C. burnetii*, such as SNP genotyping [36], 16S sequencing [37], or IS*1111*-based genotyping [38].

It is not known how the genotypes defined by the different methods described above correlate with each other, or how these genotypes can be placed within genomic groups. Therefore, we collated genotyping data from various sources and created a combined database allowing us to address this question. We also analysed the associated metadata, including the geographic distribution of genomic groups. Pooling and comparing data obtained in different laboratories enables interrogation of a larger dataset, thereby providing greater statistical power to the analyses of the relationships of genotypes with clinical outcomes, host sources, and geographical origins. Moreover, rapid genomotyping, i.e., assigning an isolate to a genomic group based on a simple assay, could help to identify the source of Q fever infections and to estimate the virulence of any new isolates.

## 2. Results

### 2.1. Clustering of MLVA Genotypes into Genomic Groups

**Figure 1:**
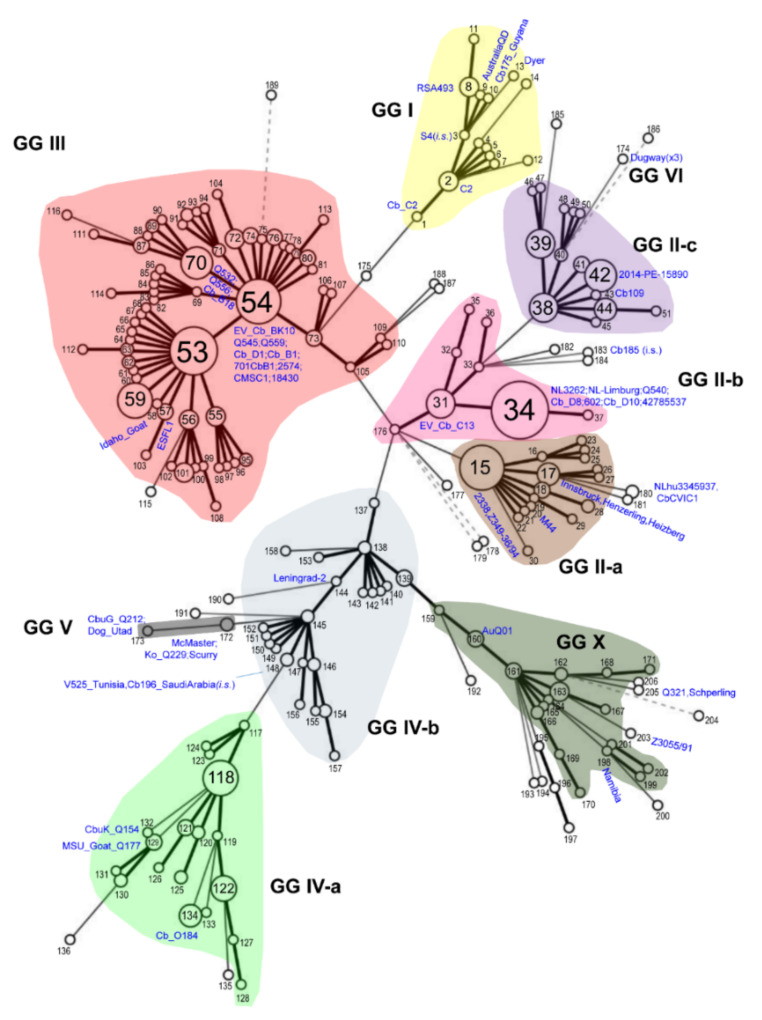
Minimum spanning tree of MLVA genotypes clustering into genomic groups. Each circle represents a unique genotype, where the size of the circle corresponds to the number of entries with the same genotype. Numbers correspond to the genotypes in Supplementary Data File Table S1A,B. Thick, short lines connecting two genotypes denote differences in a single locus; thin, longer lines connect double-locus variants; and dashed lines indicate the most likely connection between two types differing in more than two loci. Genomic groups are shaded and boundaries were defined by genotypes connected by a thick line. Whole-genome sequenced isolates are shown in blue.

We firstly analysed an MLVA dataset of 1044 entries (see Materials and Methods for details). We performed UPGMA clustering and created a minimum spanning tree. Entries fell into 206 different MLVA genotypes (see Supplemental Data File S1B). Figure 1 shows the clustering of MLVA genotypes and how these correlate with genomic groups, using whole-genome sequenced isolates as reference points. The GG II isolates fell into three distinct clusters, which we named GG II-a to -c. We previously reported the distinction of GG II-a (MST18 and 25-like) and GG II-b (MST33-like) based on core SNPs and genome content [21], whereas the third cluster, GG II-c, contained sequenced strains which we had previously determined to represent the MST32 genotype. GG IV isolates also fell into three clusters, which we named GG IV-a, GG IV-b and GG X, with the latter containing Australian isolates [20].

**Figure 2:**
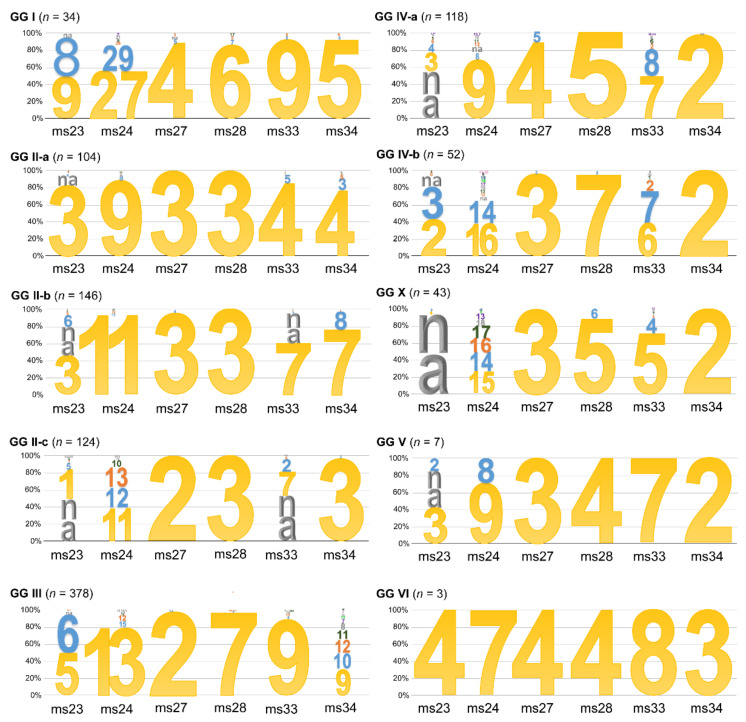
Proportional representation of allele numbers at six VNTR loci in different genomic groups. Entries had been assigned to genomic groups as seen in Figure 1. Colours were assigned by frequency, with yellow indicating the most frequently found allele number per genomic group. na = no data available.

Next, we used diversity index calculations to corroborate our assignment of MLVA genotypes into genomic groups. For this, we looked for a reduction in diversity as an indicator of categorisation. A graphic view of the resulting indices is shown in Figure S1. The diversity indices of combined database entries was between 0.62 and 0.87, whereas the indices for ten loci within genomic groups were zero, confirming uniformity. When comparing the VNTR profiles between genomic groups, we found unique, identifying repeat numbers for at least one locus for each genomic group, as well as hyper-variability in other loci (see Figure 2). All GG II entries had a repeat number of 3 for the ms28 locus, and subgroups a-c were clearly distinguishable at either the ms27 locus (a repeat number of 2 identifies GG II-c) or the ms34 locus. GG III can be identified at locus 27 (repeat number 2 in 99.7% of entries) and locus 28 (repeat number 7 in 98.3% of entries), as well as either 5 or 6 repeats at the ms23 locus. GG IV as a whole is characterised by two repeats at the ms34 locus, whereas subgroups could be identified through the combination of repeat numbers at loci ms27 and ms28.

Finally, we visualised the metadata associated with the MLVA database by colouring the minimum spanning tree (Figure 1) according to various attributes such as predicted genomic group, associated MST genotype or host and geographical isolation source. As seen in Figure S2b–d, the cluster of MLVA genotypes assigned to GG III corresponded to the MST20 genotype, which was associated mainly with cattle from Europe. A cluster of MLVA genotypes assigned to GG II-b corresponded to the MST33 genotype, which was dominated by human and goat isolates from the Netherlands and other regions of Europe. The cluster of MLVA genotypes assigned to GG IV-a contained MST8 and related genotypes that were isolated from sheep, goats or humans. The cluster of MLVA genotypes assigned to GG IV-b was represented by MST genotypes 1–7 and predominantly included isolates from humans.

### 2.2. Clustering of MST Genotypes into Genomic Groups

Next, we established the phylogenetic relationship of 66 different MST genotypes (see Supplemental Data File S1C). Genomic groups were assigned using whole-genome sequenced reference isolates as anchor points. A PhyML tree shown in Figure S3a confirmed the existence of GG IV-a as a sub-branch in the phylogenetic tree. The remaining genotypes of GG IV, which we grouped into GG IV-b, showed a low degree of clustering. The PhyML tree showed a clear distinction between GG II-a (MST18/25-like) and the remaining genotypes of GG II, but did not reveal the GG II-c cluster seen by MLVA genotyping. We, therefore, investigated whether a similarity-based method could be used to investigate the grouping of genotypes and clustering into genomic groups without assessing the phylogenetic relationship per se (see Methods for details). The resulting dendrogram (Figure S3b) shows a similar pattern of grouping as the PhyML tree, but revealed sub-branches corresponding to GG II-b (MST33-like) and GG II-c (MST32-like), as well as a third cluster here termed GG II-d (MST62–65).

To assess why a sequenced-based phylogeny failed to separate GG II-b and GG II-c isolates, which clearly have distinct MLVA profiles (see Figure 2), we aligned all available sequences for each spacer region, analysed the SNP pattern and searched for conserved SNPs that were present in >2 genotypes. These conserved SNPs commonly occurred in either GG I-III, or GG IV, which is the most distinct genetic lineage [21], whereas GG V and GG VI alleles would align with either of these lineages (see Figure S4). Using this information, we looked at each individual allele in all 66 genotypes in order to test for the presence of a GG I-III or GG IV(-VI) SNP(s), and found 19 instances where an allele did not possess the conserved SNP for the assigned genomic group (see Table 1 and Supplemental Data File Table S1C). A total of 17 out of 19 genotypes with such aberrant alleles only had a single representative in the MST database. Our final interpretation of the placement of MST genotypes into genomic groups after exclusion of these aberrant alleles, which matches the results of the similarity-based clustering, is shown in Figure S5a and Table 1.

Next, the metadata of an MST database containing 638 *C. burnetii* entries (see Supplemental Data File Table S1D) were analysed as above. A total of 434 (68%) of the entries were European isolates. Similarity-based clustering was performed as described for the individual MST genotypes, and the results were visualised as a minimum spanning tree. The plain tree depicting the MST genotype of each cluster is shown in Figure S5a, whereas Figure S5b shows how these correlate with genomic groups. Visualisation of the host source (Figure S5c) and the geographical isolation source (Figure S5d) of each entry matched the results seen using MLVA genotyping data shown in Figure S2, and confirmed that both GG II and GG IV-b have not been isolated from North America. Notably, most of the entries that were isolated outside of Europe, North America, or Central Asia, formed distinct clusters. For example, GG II-d (MST62–65) has only been isolated in Iran, GG I-b (MST17) has only been isolated in French Guyana, GG IX (MST30) and GG X (MST-AUST) have only been isolated in Namibia and Australia, respectively, and a group comprising MST66–70 has only been isolated in Thailand.

### 2.3. Analysis of A Combined Metadata Database

Since visualisation of the host source of the isolates included in the MLVA or MST databases revealed similar trends, we created a combined MST and MLVA metadata-set (see Supplemental Data File Table S1E) in order to increase the statistical power of metadata analysis. The resulting dataset contained 1434 entries assigned to a genomic group.

**Figure 3:**
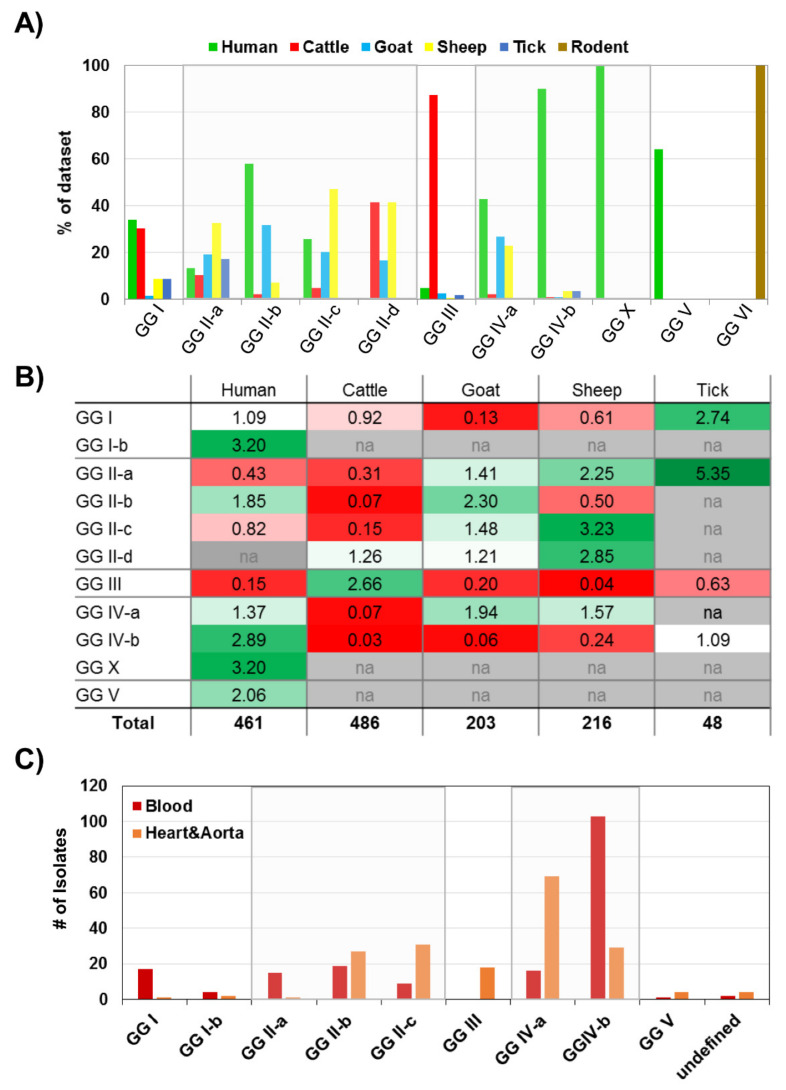
Distribution and associations of host isolation source and genomic groups. A combined metadata database containing 1475 entries (1434 of which could be assigned to a genomic group) was analysed. (**A**) Relative distribution of host sources in percentage of total entries in subpanel of entries isolated from the six host organisms shown. (**B**) Ratios of observed counts/expected counts of a Pearson’s chi-square test were calculated and colour coded. Red = lower than expected incidence; green = higher than expected incidence. Note that ratios were calculated for all entries and all host sources, but only a subset of the five most abundant animal host sources are shown. (**C**) Subset analysis of human isolates only that were either obtained from blood (*n* = 186), or from heart valve/prosthesis and aortic tissue (*n* = 186), representing 81% of the total dataset (see red and green data points in Figure S6), and their association with genomic groups. Note that GG IV-b here includes GG X.

Firstly, we calculated the frequencies of different isolation sources for each genomic group. As seen in Figure 3a, 90% and 100% of isolates belonging to GG IV-b or GG X had been isolated from cases of human disease, respectively, and 58% and 43% of isolates belonging to GG II-b or GG V had been recovered from humans, respectively. The percentages in all other genomic groups were below 40%.

Next, we performed a cross-tabulation coupled with a Pearson’s chi-square test for independence, with ‘genomic group’ as one variable and ‘host’ as the second variable. The *p* value for the chi-square test was highly significant (*p* < 0.001), suggesting that the association of ‘host’ isolation source and genomic groups was not random. Ratios of observed vs. expected counts were used to test for over- and under-representation of certain host sources in each genomic group (see Figure 3b). Cattle samples were over-represented in GG III, whereas goat, sheep, and human samples were under-represented. Differences between the host association within sub-lineages of GG II and GG IV were also apparent: human isolates were under-represented in GG II-a and II-c, but were over-represented in GG II-b, which includes the Netherlands outbreak genotype MST33. Caprine isolates were over-represented in GG II-b, but ovine isolates were under-represented. Human-derived isolates were under-represented in GG IV-a, which included the MST8 genotype, compared to GG IV-b and the linked sub-branch GG X. MST8 is the most commonly found genotype in goats milk in the US [7], and we also found an association of caprine isolates with GG IV-a.

We next analysed database entries with “human” as the isolation source (*n* = 458). As seen in Figure 3c, the absolute numbers of human isolates were highest in GG II-b, GG IV-a and GG IV-b (95, 93 and 101 entries, respectively). Human isolates in GG II-a had predominantly been isolated from blood, whereas entries belonging to GG II-b and GG II-c were frequently isolated from tissue. Isolates assigned to GG IV-b and GG X had been isolated from blood more frequently than from tissues, whereas the reverse was true for isolates in GG IV-a. A proportional representation of the results, including other sample types, is shown in Figure S6 in the Supplementary information.

**Figure 4:**
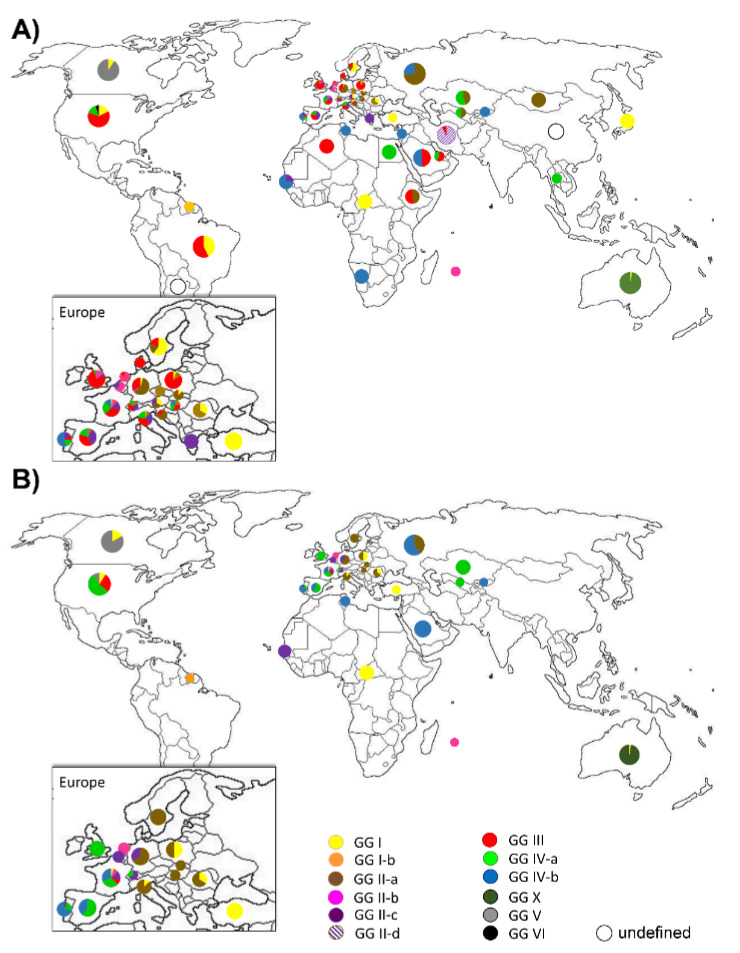
Geographic distribution of genomic groups. (**A**) All entries of a combined MLVA and MST dataset (*n* = 1475, 1434 of which were assigned to a genomic group). (**B**) Human isolates only of the same combined dataset (*n* = 461). In A&B, Pie charts show the relative abundance of each genomic group per country. Note that a central location within the boundaries of each country was chosen.

Finally, we also assessed the geographic origins of isolates in each genomic group. As seen in Figure 4a, GG I isolates have been found on every continent. GG II-a isolates have been found predominantly in Northern and Central Asia, Eastern and Central Europe and Africa. Isolates assigned to GG II-b originate predominantly in the Benelux and neighbouring countries, including the United Kingdom. GG II-c isolates are found in Western and Southern Europe, whilst isolates in GG II-d originate from Iran. GG III is frequently found in Europe, the Americas, and Africa, but has not yet been reported in Australia or Northern and Central Asia. GG IV-a has been found in Southern and Central Asia, the Middle East, Southern Europe, and North America. GG IV-b is predominant in Northern and Central Asia, the Middle East and Africa, as well as some Western and Central parts of Europe, especially Portugal. GG V and VI are restricted to North America.

When re-drawing the map with only entries with a human isolation source (see Figure 4b), GG III almost entirely vanished from the map, whereas GG IV predominated Western Europe and Asia and GG II-a predominated Northern, Central, and South-eastern Europe. Both GG II-b and II-c clustered around France and Germany of Central Europe.

### 2.4. Correlation between 16S and SNP Genotypes and Genomic Groups

We also assessed other, less frequently reported genotyping methods, for their abilities to assign isolates into genomic groups. Firstly, we extracted the 16S sequences of all whole-genome sequenced isolates which we have previously assigned into a genomic group [21] and compared their SNP profile relative to the Nine Mile reference strain. As shown in Figure S7a, SNPs were found at eight positions in the 16S coding sequence. Unique identifying SNPs were found for GG II-a, GG IV-a, GG V, and GG I-b and GG IV. However, GG I, GG III, and GG VI were indistinguishable. Next, we assessed SNP genotyping after extracting seven SNP regions described by Huijsman et al. [36] from genome sequences. Unique identifying SNPs were found for GG II-a, GG V, GG IV and GG II (Figure S7b). As seen with 16S genotyping, GG I, GG III, and GG VI were indistinguishable.

### 2.5. Establishing a Novel Genomotyping Method Based on the Cox51 Spacer Sequence

**Figure 5:**
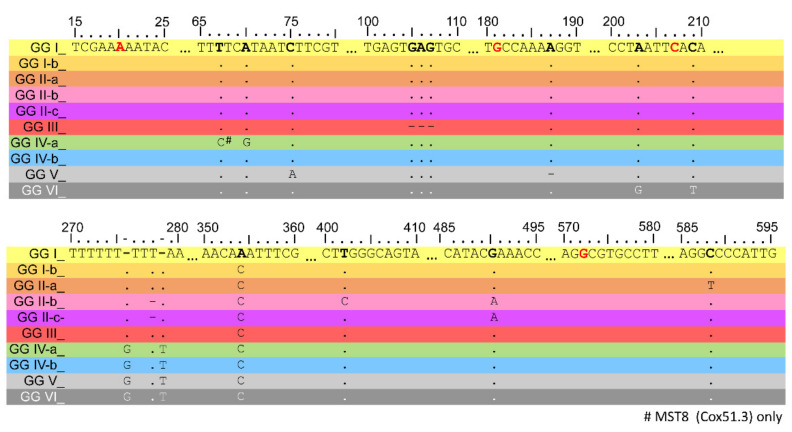
Genomotyping SNP scheme for the Cox51 spacer region. Sequences for each genomic group were aligned and SNPs relative to the Nine Mile RSA493 reference genome belonging to GG I are shown. Dots represent no change, whereas dashes represent a deletion. Unique, identifying SNPs were found for GG I, I-b, II-a, II-b, III, IV-a, V, and VI. GG II-c and d are indistinguishable from each other, but can be distinguished from GG II-b by the absence of a SNP at position 403. GG IV-b can be deduced by the presence of the common GG IV-VI insertions (pos 276 and 278) without the presence of the identifying SNPs for GG IV-a, V, and VI. Nucleotide positions where SNPs have occurred in isolates that were not conserved within a genomic group are highlighted in red. Note that GG IV-b includes GG IX and GG X (MST30 and MST-Aust).

Finally, we asked whether any of the spacer sequences used in MST genotyping would be suitable for the rapid assignment of an isolate to a genomic group. For this, we sorted all 66 MST genotypes depending on their genomic group (see Supplemental Data File Table S1C) and looked for alleles conserved in abundance in each genomic group. Only the Cox51 spacer allele showed such a conservation (Table 2), and a sequence alignment of these alleles showed unique, identifying SNPs for individual genomic groups or related lineages, with a clear split between GG I-III and GG IV-VI (see Figure 5). In cases where a genomic group contained multiple alleles (Table 2), all of these alleles shared the unique, identifying SNPs for this GG, but with additional SNPs being present (data not shown). In summary, we propose that sequencing the Cox51 intergenic region after PCR amplification using the primers described by Glazunova et al. [30] could represent a quick and inexpensive method for genomotyping of *C. burnetii*.

## 3. Discussion

In this study, we aimed to correlate MST and MLVA genotyping data for *C. burnetii* isolates with their genetic lineage, i.e., genomic group. Previous studies have assessed various attributes of individual genotypes such as their geographic location [39], but no such data exist for genomic groups. There is growing evidence of different virulence potential between different genomic groups [22] and our comparison of the makeup of different genomic groups [21] revealed few genetic differences between isolates belonging to the same lineage. Therefore, we argue that a comparison of genomic groups with almost identical gene contents, rather than individual genotypes that have been identified by differences in non-coding regions of the chromosome, is more meaningful.

We found identifying MLVA repeat numbers for all genomic groups described to date (see Figure 2), which should allow new genotypes to be assigned to a genomic group. We also assigned all known MST genotypes to a genomic group (see Table 1) and report a method for assigning isolates using the Cox51 spacer sequence (see Figure 5 and Table 2). Interestingly, most isolates from outside of Europe, N. America or Central Asia formed unique clusters with additional SNPs present. This suggests that the genetic diversity of the species is underestimated, and that the testing of additional isolates from areas outside of Europe, N. America or Central Asia is likely to yield novel genotypes and genomic groups.

Assigning MST genotypes to genomic groups using two different methods yielded broadly similar results (see Figure S3), and by combining both the MLVA and MST clustering data, we propose that GG II is divided into four sub-lineages, namely GG II-a (MST18/25), GG II-b (MST33-like), GG II-c (MST32-like), and GG II-d (MST62–65, all from Iran). The latter group had no genome-sequenced representatives or matching MLVA profile. We could explain most of the differences between the sequence-based phylogeny and the similarity-based clustering by the presence of seemingly aberrant sequences in 19 genotypes (see Table 2 and Supplemental Data File S1C) typically involving genotypes with one representative isolate. This might be a result of sequencing errors, or a mixture of genotypes being present in these samples, or it might be a real phenomenon. More widespread sampling in geographical regions where these genotypes have been isolated or whole-genome sequencing of these genotypes is required to resolve this issue of aberrant alleles.

As expected, MLVA genotyping was more sensitive than MST genotyping, with over 200 genotypes observed. Some entries had missing data points, most frequently at the ms23 and ms33 loci, both of which contain recognition sites for the IS*1111* insertion element [40]. We also observed misplacement of some isolates in the MLVA minimum spanning tree (Figure 1). Most of these misplaced entries were in silico MLVA typed using an online algorithm and manual curation, where necessary. It is possible that missassembly of sequencing reads over the repeated regions resulted in an aberrant repeat number. This included MLVA genotype 180 containing in silico genotyped entries NLhu3345937 and CbCVIC1, both of which have been shown to be MST33 and assigned to GG II-b by whole-genome sequencing, but which were placed next to GG II-a. Isolate Q321 is MLVA genotype 205 and although a previous microarray analysis suggested it was most closely related to GG IV isolates [19], multiple unique polymorphisms led the authors to suggest that Q321 should be classed into genomic group VII. However, our data suggest that GG VII is part of GG IV-b. Genotype 183 has been defined as MST18, and thus as GG II-a. However, the MLVA profile placed it between GG II-b and GG II-c, alongside genotypes 182 and 184. These isolates might represent novel sub-lineages but additional genome sequences would be required to confirm this. Finally, genotype 130 is represented by four isolates from Hungary described as MST37 [41]. The MLVA profile matches GG IV-a (see Figure 2) but sequence- and similarity-based methods place MST37 in GG IV-b (see Figure S5), and the genotype has a Cox51.8 allele indicative of GG IV-b. The reason for this discrepancy remains unclear.

Overall, there was good correlation between genomic groups assigned via MST or MLVA genotyping. However, we did not observe strict clustering of MST genotypes within MLVA genotypes, but these were distributed across multiple MLVA genotypes within a genomic group (see Figure S2b). This finding supports the hypothesis that different regions of the chromosome evolve at different rates. It also supports our suggestion that assigning an isolate to a genomic group (genomotyping) rather than individual genotypes, is more meaningful, and Cox51 allele diversity allows such rapid genomotyping. Individual SNPs in the Cox51 and other MST loci have previously been used to perform SNP genotyping of individual MST genotypes [42], but not genomic groups on the whole.

There is growing evidence of different host tropisms and disease potentials of different genomic groups. Different animal models have been used to assess the virulence of *C. burnetii* isolates, and we have recently reviewed the genomic group-specific virulence data [22] although it is not clear how virulence in animals relates to virulence in humans. Most importantly, GG II and III isolates were under-represented in these studies, and no direct comparisons of sub-lineages of GG II and IV were performed. For instance, the study by Long et al. [43] performed in guinea pigs only used one GG II isolate (strain Henzerling, MST18 of GG II-a), and both GG IV isolates, MSU_Goat (Priscilla) and P_Q238, used in the study have been described as MST8 of GG IV-a [44]. Russel-Lodrigue et al. [45] also tested strain MSU_Goat, as well as a third GG IV isolate, P_Q173, in guinea pigs and mice. P_Q173 shares a similar genome content as MSU_Goat in a microarray study [19] and is, therefore, also considered to belong to GG IV-a; however, both strains showed slight differences in signs of disease. Recent virulence data have been published for two GG II-b isolates, namely Z3055 and CbBEC1 (both MST33) in both immunocompetent and immunocompromised mice [46,47], and both were more virulent than the Nine Mile reference strain of GG I, at least when splenic involvement was assessed. In the study by Melenotte et al. [46], isolate Cb175_Guyana (MST17) was more virulent than strain Z3055 or the Nine Mile strain. The MST17 genotype differs from MST16 isolates by more than 300 SNPs [21,46] and we have previously proposed that this genotype is a sub-lineage of GG I [21], here termed GG I-b.

When considering only human disease isolates, we found that these were over-represented in GG II-b, IV-b, V and X. This can partly be explained by sampling biases, such as during the Q fever outbreak in the Netherlands for GG II-b, and the lack of environmental sampling in Australia for GG X. Worldwide, representatives from all but GG III and GG VI have been isolated from humans (see Figure 4b), but there seem to be regional differences in the predominant disease causing lineages. The observation that closely related GG II-b and c isolates as well as GG IV-a isolates were more frequently found in tissue samples (see Figure 3c), which might indicate chronic Q fever complications such as endocarditis, and that GG II-a isolates as well as GG IV-b isolates were more frequently isolated from blood samples, which most likely indicates acute Q fever due to the transient nature of *C. burnetii* bacteraemia, is corroborated by results from a study by Glazunova et al. [30], which also found that acute Q fever was associated with genotypes MST1, and MST4 (both GG IV-b), MST16 (GG I), and MST18 (GG II-a), whereas chronic Q fever was associated with MST8 (GG IV-a). In animal models, it has been shown that both MST33 (GG II-b) and MST8 (GG IV-a) isolates tend to cause a more focalised infection [43,47], and persistent focalised infections also occur in humans and are often labelled as chronic Q fever [48]. Older studies have also observed that isolates that contain plasmid QpRS (later shown to be specific to GG IV) are supposedly linked with persistent focalised infection [8]; however, genotyping data are only available for some of the isolates used in the study, and all of the GG IV isolates were found to belong to MST8. It is, therefore, possible that studies that formed the initial distinction between acute and chronic isolates had only included GG IV-a isolates, and that the theory was later discarded when other GG IV-b isolates, which can also contain QpRS, were included without realising the chromosomal differences between the sub-lineages in addition to their plasmid content. However, since no true chronic animal model is available to date, the common theory that GG IV-a isolates might cause more chronic infections remains largely untested.

In summary, the dataset described in this study allows us to draw preliminary conclusions about associations between genetic makeup, host tropism and disease severity, even though the exact genetic factors remain to be elucidated. More animal studies, including the development of a chronic animal model, would be required to confirm the proposed differences between sub-lineages of GG II and GG IV. Moreover, broader sampling of both animal and clinical samples in a broader range of countries, especially in non-European/non-North American countries, as well as data deposition in public databases would be required to overcome the sampling biases in the current dataset. Finally, obtaining genotyping data, or even whole-genome sequencing (for instance using culture-independent sequencing methods like the one described by us [21]) for every outbreak of Q fever would improve the predictive power of genomotyping and, therefore, any such efforts are greatly encouraged.

## 4. Materials and Methods

### 4.1. Establishment of MST Genotypes

The MST genotype database (https://ifr48.timone.univ-mrs.fr/mst/coxiella_burnetii/strains.html accessed on 4 November 2020) hosted by the Méditerranée-Infection Institute contained 69 different MST genotypes, numbered MST1 to MST74, at the time of writing; no data were present for five genotypes (MST14, 26, 34, 52, 56), and another five genotypes (MST35, 36, 58, 59, 74) were excluded as they only contain data for ≤6 out of ten spacer regions. Two genotypes, MST51 and MST57, contain data for nine and eight out of ten spacer regions, respectively, which were all included in the analysis. MST60, which we found to share nine out of ten spacer alleles with the Dugway strains [25], contained an allele, Cox56.14, which, together with Cox56.13, did not align with any of the other Cox56 sequences and we, therefore, only used in silico-generated sequences from the Dugway isolates (MST-DUG) for the phylogenetic analyses. We also included in silico-generated allele sequences for a whole-genome sequenced strain, isolate AuQ01 from Australia [49], which showed novel alleles and allele combinations that were not included in the MST database and which we termed genotype “AUST”. We also inferred that MST genotypes 71 and 72 correspond to two isolates from hedgehogs in China [50] and, therefore, added the metadata for these two genotypes into our database. Lastly, we also included a novel genotype from a recent publication, namely isolate EG11 from Egypt [51], which we subsequently termed EGYPT. This resulted in a final database of 66 different MST genotypes.

### 4.2. Phylogenetic Analysis of MST Genotypes

The spacer sequences of each of the 66 MST genotypes were concatenated and aligned using the SeaView alignment editor. A PhyML tree was created from variable sites in the SeaView alignment using 500 × bootstrap iterations, and trees were analysed in FigTree graphical viewer. This included rooting of the tree along the branch leading to what we perceived as GG IV [26]. The similarities between allele numbers of the same genotypes were also analysed using the Bionumerics software (AppliedMaths), as described below for the whole MST database. For the comparison of alleles for each individual spacer regions, allele sequences were aligned using the MegaX software [52] and SNPs that occurred in more than two alleles were used to order the sequences. The occurrence of these alleles containing conserved SNPs in different genetic lineages was analysed by calculating the frequencies of these alleles in each genomic group. This allowed us to distinguish SNPs that either indicated the GG I–III group, or the GG IV group (either as a whole or GG IV-a specific). GG V and GG VI alleles aligned with either of these lineages or stayed separate.

### 4.3. Establishment of MST and MLVA Allele and Metadata Databases

MST and MLVA profiles including metadata such as isolation source (host species and geographical location) were extracted from published journal articles, as well from online databases. The MST strain database contained 329 strain entries, which was complemented with additional entries from data included in publications referenced here [10,51,53,54,55,56,57,58,59,60,61,62,63,64,65,66,67] to make up a final database with 638 entries in total (see Supplemental Data File S1D). The MLVA database contained 379 entries at time of writing (http://microbesgenotyping.i2bc.paris-saclay.fr/databases/view/966 accessed on 9 April 2020). Only the MLVA6Nijmegen panel was considered as it is the most commonly used panel. The panel contains three hexanucleotide repeat markers (Ms27, Ms28 and Ms34), and three heptanucleotide repeat markers (Ms23, Ms24 and Ms33) [34,68]. The database was complemented with additional entries from data included in publications referenced here [20,32,34,47,53,58,66,67,69,70,71,72,73,74,75,76]. Where isolates did not have a unique identifier, consecutive numbers were assigned as by order in the publication (see Supplemental Data File S1A).

### 4.4. Analysis of MST and MLVA Allele and Metadata Databases

The MST (638 entries) and MLVA lists (1044 entries) were imported as character data into the Bionumerics software (AppliedMaths), where MST data were treated like MLST data. For this, a similarity matrix of categorical values (allele numbers) was used, which does not quantify differences in allele numbers (and, therefore, does not test for phylogenetic relatedness per se), but simply assesses whether allele numbers are the same or different for each spacer region. Both datasets were compared using the UPGMA (Unweighted Pair Group Method with Arithmetic mean) clustering method to generate minimum spanning trees showing relationships between genotypes. Metadata were analysed using IBM SPSS Statistics (v26) using crosstabs and chi-square statistical tests. To assess the discriminatory power of the MLVA method for samples analysed in this study, Simpson’s diversity indices and corresponding 95% confidence intervals were calculated for each tested locus and for the overall MLVA method using the online tool Comparing Partitions (http://www.comparingpartitions.info/?link=Tool accessed on 6 June 2020), which also provided histogram data used to create logos of repeat numbers in each genomic group.

## Figures and Tables

**Table 1:** MST genotypes per genomic group. MST genotypes in brackets have at least one aberrant allele present that contains at least one SNP that is conserved in divergent lineage(s). Genotypes that have only one representative are indicated in italics. The most frequently overserved genotype per GG is indicated in bold.

Genomic Group	MST Genotype
GG I	**16**
GG I-b	**17**
GG II-a	**18**, 22, 23, 25 (*29*)
GG II-b	**33** (*15*, *44*, *46*)
GG II-c	11, 12, **13**, *24*, 32, *40* (*39*, *47*)
GG II-d	**62**, *63*, 64, *65*
GG III	**20**, 61
link (GG I-III like)	*19*, *49*
GG IV-a	**8**, 9, 10, *27*, 28, *31*, *50*, *53*, 55, *66*, *68*, *69*, *70*, *EGYPT* (38, 42, *43*, *48*, *51*, *54*, *67*)
GG IV-b	**1**, 2, *3*, 4, 5, 6, 7, *30*, *Aust* (37, *41*, *45*, *57*)
GG IV-b associated	*71 (72*, *73)*
GG V	**21**
GG VI	**60**

**Table 2:** Proposed Cox51 genomotyping scheme.

Genomic Group	Representative MST Genotypes	Cox51 Allele
GG I	16	Cox51.6
GG I-b	17	Cox51.10
GG II-a	18, 22, 23, 25, 29	Cox51.7
GG II-b	15, 33, 44, 46	Cox51.9
GG II-c and d	11–13, 24, 32, 39, 40, 47, 62–65	Cox51.5
GG III	20, 61	Cox51.4
link (GG I–III like)	19, 49	Cox51.11
GG IV-a	42, 51, 54, 55, EGYPT	Cox51.2
	8, 38, 43, 48, 50, 53	Cox51.3
	67, 69, 70	Cox51.14
	66, 68	Cox51.15
GG IV-b	1–7, 30, 37, 41, 45, 72, AUST	Cox51.8
GG IV-b associated	71	Cox51.16
	73	Cox51.17
GG V	21	Cox51.1
GG VI	DUG (60)	Cox51.12
unassigned	no representative	Cox51.13

## Data Availability

All data are available in the Supplementary data file.

## Supplementary Materials

The following are available online at https://www.mdpi.com/article/10.3390/pathogens10050604/s1, Figure S1: Diversity indices of six VNTR regions for various groups of MLVA genotypes, as calculated using the online tool “Comparing Partitions”. Diversity indexes were calculated for all entries or for subgroups of entries as seen in the legend. A diversity index of zero signifies complete uniformity. Figure S2: Minimum spanning tree of MLVA database entries (*n* = 1044) colour coded by genomic group, as predicted based on allele number profiles seen in Figure S2 (A), associated MST genotypes (B), host isolation source (C), and geographical isolation source (D). Figure S3: Phylogenetic relationship and clustering of MST genotypes, and association with genomic groups. (A) Unrooted PhyML tree of concatenated spacer sequences using 500 bootstraps. (B) Dendrogram of similarity clustering of MST allele numbers using UPGMA. Genomic Groups were termed in line with Figure S2b. Note that isolates belonging to MST2 (Q321, Schperling) are sometimes referred to as GG VII in the literature. Figure S4: Shared SNPs within MST spacer sequence alignments. All available alleles for each spacer region were aligned and positions with SNPs in ≥2 sequences are shown. The alleles were cross-referenced with our MST genotype database to identify SNPs that were unique for a genomic group or groups of GGs. Note that the position refers to the alignment, not individual sequences. Figure S5: Minimum spanning trees of MST genotypes and visualisation of associated metadata for 638 database entries. Each circle represents a unique genotype, and each segment of a circle a single isolate. Thick, short lines connecting two genotypes denote differences in a single locus; thin, longer lines connect double-locus variants; and dashed lines indicate the most likely connection between two types differing in more than two loci. (A) plain view of MST genotypes; (B) coloured view based on predicted genomic group (see Figure S3b); (C) coloured view based on isolation source; (D) coloured view based on geographic isolation source (by continents). Figure S6: Proportional abundance of each human sample type per genomic group in a subset including only entries with “human” as isolation source. Figure S7: Correlation of alternative genotyping methods with genomic groups, as determined through in silico analyses of 67 published genomes. (A) 16S genotyping and genomic groups. The full-length 16S coding sequences representing each genomic group were compared to the GG I (RSA493) sequence, and the position of each SNPs is shown. Note that GG X is included in GG IV-b (B) SNP genotyping and genomic groups. The SNP profile of each genomic group at the position noted at the top of the figure (within the RSA493 reference genome) was compared and correlated to the SNP genotype associated with each profile. Supplementary Data File: MST and MLVA databases.

## References

[1] Million M., Raoult D. (2015). Recent advances in the study of Q fever epidemiology, diagnosis and management. J. Infect..

[2] Honarmand H. (2012). Q Fever: An Old but Still a Poorly Understood Disease. Interdiscip. Perspect. Infect. Dis..

[3] Maurin M., Raoult D. (1999). Q Fever. Clin. Microbiol. Rev..

[4] Pexara A., Solomakos N., Govaris A. (2018). A review on the seroprevalence of *Coxiella burnetii* in farm ruminants in various countries. Vet. Ital..

[5] Karagiannis I., Schimmer B., Van Lier A., Timen A., Schneeberger P., Van Rotterdam B., De Bruin A., Wijkmans C., Rietveld A., Van Duynhoven Y. (2009). Investigation of a Q fever outbreak in a rural area of The Netherlands. Epidemiol. Infect..

[6] Anderson A.D., Bijlmer H.A., Fournier P.-E., Graves S.E., Hartzell J.D., Kersh G.J., Limonard G.J.M., Marrie T.J., Massung R.F., McQuiston J.H. (2013). Diagnosis and management of Q fever—United States, 2013: Recommendations from CDC and the Q Fever Working Group. Morb. Mortal. Wkly. Rep. Recomm. Rep..

[7] Angelakis E., Raoult D. (2010). Q fever. Vet. Microbiol..

[8] Samuel J.E., Frazier M.E., Mallavia L.P. (1985). Correlation of plasmid type and disease caused by *Coxiella burnetii*. Infect. Immun..

[9] Hendrix L.R., Samuel J.E., Mallavia L.P. (1991). Differentiation of *Coxiella burnetii* isolates by analysis of restriction-endonuclease-digested DNA separated by SDS-PAGE. Microbiology.

[10] Hornstra H.M., Priestley R.A., Georgia S.M., Kachur S., Birdsell D.N., Hilsabeck R., Gates L.T., Samuel J.E., Heinzen R.A., Kersh G.J. (2011). Rapid Typing of *Coxiella burnetii*. PLoS ONE.

[11] Duron O., Noël V., McCoy K.D., Bonazzi M., Sidi-Boumedine K., Morel O., Vavre F., Zenner L., Jourdain E., Durand P. (2015). The Recent Evolution of a Maternally-Inherited Endosymbiont of Ticks Led to the Emergence of the Q Fever Pathogen, *Coxiella burnetii*. PLoS Pathog..

[12] Robbins F.C., Rustigian R., Snyder M.J., Smadel J.E. (1946). Q Fever in the mediterranean area: Report of its occurrence in allied troops. Part III: Etiological Agent. Am. J. Epidemiol..

[13] Topping N.H., Shepard C.C., Huebner R.J. (1946). Q fever; an immunological comparison of strains. Am. J. Epidemiol..

[14] Stoker M.G.P. (1950). Q Fever in Great Britain: The causative agent. Lancet.

[15] Stoenner H.G., Lackman D.B. (1960). The biologic properties of *Coxiella burnetii* isolated from rodents collected in Utah. Am. J. Epidemiol..

[16] Burnet F.M., Feeeman M. (1939). A comparative study of Rickettsial strains from an infection of ticks in Montana (United States of America) and from “Q” Fever. Med. J. Aust..

[17] Jäger C., Willems H., Thiele D., Baljer G. (1998). Molecular characterization of *Coxiella burnetii* isolates. Epidemiol. Infect..

[18] D’Amato F., Eldin C., Raoult D. (2016). The contribution of genomics to the study of Q fever. Future Microbiol..

[19] Beare P.A., Samuel J.E., Howe D., Virtaneva K., Porcella S.F., Heinzen R.A. (2006). Genetic Diversity of the Q Fever Agent, *Coxiella burnetii*, Assessed by Microarray-Based Whole-Genome Comparisons. J. Bacteriol..

[20] Vincent G., Stenos J., Latham J., Fenwick S., Graves S. (2016). Novel genotypes of *Coxiella burnetii* identified in isolates from Australian Q fever patients. Int. J. Med. Microbiol..

[21] Hemsley C.M., O’Neill P.A., Essex-Lopresti A., Norville I.H., Atkins T.P., Titball R.W. (2019). Extensive genome analysis *of Coxiella burnetii* reveals limited evolution within genomic groups. BMC Genom..

[22] Metters G., Norville I.H., Titball R.W., Hemsley C.M. (2019). From cell culture to cynomolgus macaque: Infection models show lineage-specific virulence potential of *Coxiella burnetii*. J. Med. Microbiol..

[23] Kuley R., Kuijt E., Smits M.A., Roest H.I.J., Smith H.E., Bossers A. (2017). Genome Plasticity and Polymorphisms in Critical Genes Correlate with Increased Virulence of Dutch Outbreak-Related *Coxiella burnetii* Strains. Front. Microbiol..

[24] D’Amato F., Rouli L., Edouard S., Tyczka J., Million M., Robert C., Nguyen T.T., Raoult D. (2014). The genome of *Coxiella burnetii* Z3055, a clone linked to the Netherlands Q fever outbreaks, provides evidence for the role of drift in the emergence of epidemic clones. Comp. Immunol. Microbiol. Infect. Dis..

[25] Beare P.A., Unsworth N., Andoh M., Voth D.E., Omsland A., Gilk S.D., Williams K.P., Sobral B.W., Kupko J.J., Porcella S.F. (2009). Comparative Genomics Reveal Extensive Transposon-Mediated Genomic Plasticity and Diversity among Potential Effector Proteins within the Genus *Coxiella*. Infect. Immun..

[26] Pearson T., Hornstra H.M., Sahl J.W., Schaack S., Schupp J.M., Beckstrom-Sternberg S.M., O’Neill M.W., Priestley R.A., Champion M.D., Beckstrom-Sternberg J.S. (2013). When Outgroups Fail; Phylogenomics of Rooting the Emerging Pathogen, *Coxiella burnetii*. Syst. Biol..

[27] Larson C.L., Martinez E., Beare P.A., Jeffrey B., Heinzen R.A., Bonazzi M. (2016). Right on Q: Genetics begin to unravel *Coxiella burnetii* host cell interactions. Future Microbiol..

[28] Li W., Raoult D., Fournier P.-E. (2009). Bacterial strain typing in the genomic era. FEMS Microbiol. Rev..

[29] Drancourt M., Roux V., Dang L.V., Tran-Hung L., Castex D., Chenal-Francisque V., Ogata H., Fournier P.-E., Crubézy E., Raoult D. (2004). Genotyping, Orientalis-like *Yersinia pestis*, and Plague Pandemics. Emerg. Infect. Dis..

[30] Glazunova O., Roux V., Freylikman O., Sekeyova Z., Fournous G., Tyczka J., Tokarevich N., Kovacova E., Marrie T.J., Raoult D. (2005). *Coxiella burnetii* genotyping. Emerg. Infect. Dis..

[31] van Belkum A., Scherer S., van Alphen L., Verbrugh H. (1998). Short-Sequence DNA Repeats in Prokaryotic Genomes. Microbiol. Mol. Biol. Rev..

[32] Svraka S., Toman R., Skultety L., Slaba K., Homan W.L. (2006). Establishment of a genotyping scheme for *Coxiella burnetii*. FEMS Microbiol. Lett..

[33] Arricau-Bouvery N., Hauck Y., Bejaoui A., Frangoulidis D., Bodier C., Souriau A., Meyer H., Neubauer H., Rodolakis A., Vergnaud G. (2006). Molecular characterization of *Coxiella burnetii* isolates by infrequent restriction site-PCR and MLVA typing. BMC Microbiol..

[34] Tilburg J.J.H.C., Rossen J.W.A., van Hannen E.J., Melchers W.J.G., Hermans M.H.A., van de Bovenkamp J., Roest H.J.I.J., De Bruin A., Nabuurs-Franssen M.H., Horrevorts A.M. (2012). Genotypic Diversity of *Coxiella burnetii* in the 2007–2010 Q Fever Outbreak Episodes in The Netherlands. J. Clin. Microbiol..

[35] Grissa I., Bouchon P., Pourcel C., Vergnaud G. MLVABank for Microbes Genotyping. http://microbesgenotyping.i2bc.paris-saclay.fr/databases/.

[36] Huijsmans C.J.J., Schellekens J.J.A., Wever P.C., Toman R., Savelkoul P.H.M., Janse I., Hermans M.H.A. (2011). Single-Nucleotide-Polymorphism Genotyping of *Coxiella burnetii* during a Q Fever Outbreak in The Netherlands. Appl. Environ. Microbiol..

[37] McLaughlin H.P., Cherney B., Hakovirta J.R., Priestley R.A., Conley A., Carter A., Hodge D., Pillai S.P., Weigel L.M., Kersh G.J. (2017). Phylogenetic inference of *Coxiella burnetii* by 16S rRNA gene sequencing. PLoS ONE.

[38] Denison A.M., Thompson H.A., Massung R.F. (2007). IS1111 insertion sequences of *Coxiella burnetii*: Characterization and use for repetitive element PCR-based differentiation of *Coxiella burnetii* isolates. BMC Microbiol..

[39] Eldin C., Mélenotte C., Mediannikov O., Ghigo E., Million M., Edouard S., Mege J.-L., Maurin M., Raoult D. (2017). From Q Fever to *Coxiella burnetii* Infection: A Paradigm Change. Clin. Microbiol. Rev..

[40] Sidi-Boumedine K., Duquesne V., Prigent M., Yang E., Joulié A., Thiéry R., Rousset E. (2015). Impact of IS1111 insertion on the MLVA genotyping of *Coxiella burnetii*. Microbes Infect..

[41] Sulyok K.M., Kreizinger Z., Hornstra H.M., Pearson T., Szigeti A., Dán Á., Balla E., Keim P.S., Gyuranecz M. (2014). Genotyping of *Coxiella burnetii* from domestic ruminants and human in Hungary: Indication of various genotypes. BMC Vet. Res..

[42] Cocking J.H., Deberg M., Schupp J., Sahl J., Wiggins K., Porty A., Hornstra H.M., Hepp C., Jardine C., Furstenau T.N. (2020). Selective whole genome amplification and sequencing of *Coxiella burnetii* directly from environmental samples. Genomics.

[43] Long C.M., Beare P.A., Cockrell D.C., Larson C.L., Heinzen R.A. (2019). Comparative virulence of diverse *Coxiella burnetii* strains. Virulence.

[44] Miller H.K., Priestley R.A., Kersh G.J. (2020). Transmission of *Coxiella burnetii* by ingestion in mice. Epidemiol. Infect..

[45] Russell-Lodrigue K.E., Andoh M., Poels M.W.J., Shive H.R., Weeks B.R., Zhang G.Q., Tersteeg C., Masegi T., Hotta A., Yamaguchi T. (2009). *Coxiella burnetii* Isolates Cause Genogroup-Specific Virulence in Mouse and Guinea Pig Models of Acute Q Fever. Infect. Immun..

[46] Melenotte C., Caputo A., Bechah Y., Lepidi H., Terras J., Kowalczewska M., Di Pinto F., Nappez C., Raoult D., Brégeon F. (2019). The hypervirulent *Coxiella burnetii* Guiana strain compared in silico, in vitro and in vivo to the Nine Mile and the German strain. Clin. Microbiol. Infect..

[47] Mori M., Boarbi S., Michel P., Bakinahe R., Rits K., Wattiau P., Fretin D. (2013). In Vitro and In Vivo Infectious Potential of *Coxiella burnetii*: A Study on Belgian Livestock Isolates. PLoS ONE.

[48] Million M., Raoult D. (2017). No Such Thing as Chronic Q Fever. Emerg. Infect. Dis..

[49] Walter M.C., Vincent G.A., Stenos J., Graves S., Frangoulidis D. (2014). Genome Sequence of *Coxiella burnetii* Strain AuQ01 (Arandale) from an Australian Patient with Acute Q Fever. Genome Announc..

[50] Gong X.-Q., Xiao X., Liu J.-W., Han H.-J., Qin X.-R., Lei S.-C., Yu X.-J. (2020). Occurrence and Genotyping of *Coxiella burnetii* in Hedgehogs in China. Vector-Borne Zoonotic Dis..

[51] Selim A., Abdelrahman A., Thiéry R., Sidi-Boumedine K. (2019). Molecular typing of *Coxiella burnetii* from sheep in Egypt. Comp. Immunol. Microbiol. Infect. Dis..

[52] Kumar S., Stecher G., Li M., Knyaz C., Tamura K. (2018). MEGA X: Molecular evolutionary genetics analysis across computing platforms. Mol. Biol. Evol..

[53] Jodełko A., Niemczuk K., Szymańska-Czerwińska M. (2015). Seroprevalence of *Coxiella burnetii* in Polish cattle herds. Bull. Veter Inst. Pulawy.

[54] Szymańska-Czerwińska M., Jodełko A., Zaręba-Marchewka K., Niemczuk K. (2019). Shedding and genetic diversity of *Coxiella burnetii* in Polish dairy cattle. PLoS ONE.

[55] Kersh G.J., Priestley R.A., Hornstra H.M., Self J.S., Fitzpatrick K.A., Biggerstaff B.J., Keim P., Pearson T., Massung R.F. (2016). Genotyping and Axenic Growth of *Coxiella burnetii* Isolates Found in the United States Environment. Vector-Borne Zoonotic Dis..

[56] Hurtado A., Alonso E., Aspiritxaga I., Etxaniz I.L., Ocabo B., Barandika J.F., De Murúa J.I.F.-O., Urbaneja F., Álvarez-Alonso R., Jado I. (2017). Environmental sampling coupled with real-time PCR and genotyping to investigate the source of a Q fever outbreak in a work setting. Epidemiol. Infect..

[57] Kumsa B., Parola P., Almeras L., Raoult D., Socolovschi C. (2015). Occurrence and Genotyping of *Coxiella burnetii* in Ixodid Ticks in Oromia, Ethiopia. Am. J. Trop. Med. Hyg..

[58] Di Domenico M., Curini V., De Massis F., Di Provvido A., Scacchia M., Cammà C. (2014). *Coxiella burnetii* in central Italy: Novel genotypes are circulating in cattle and goats. Vector-Borne Zoonotic Dis..

[59] Galiero A., Fratini F., Cammà C., Di Domenico M., Curini V., Baronti I., Turchi B., Cerri D. (2016). Occurrence of *Coxiella burnetii* in goat and ewe unpasteurized cheeses: Screening and genotyping. Int. J. Food Microbiol..

[60] Chochlakis D., Santos A.S., Giadinis N.D., Papadopoulos D., Boubaris L., Kalaitzakis E., Psaroulaki A., Kritas S.K., Petridou E.I. (2018). Genotyping of *Coxiella burnetii* in sheep and goat abortion samples. BMC Microbiol..

[61] Rahal M., Tahir D., Eldin C., Bitam I., Raoult D., Parola P., Tahir D. (2018). Genotyping of *Coxiella burnetii* detected in placental tissues from aborted dairy cattle in the north of Algeria. Comp. Immunol. Microbiol. Infect. Dis..

[62] Bauer A.E., Olivas S., Cooper M., Hornstra H., Keim P., Pearson T., Johnson A.J. (2015). Estimated herd prevalence and sequence types of *Coxiella burnetii* in bulk tank milk samples from commercial dairies in Indiana. BMC Vet. Res..

[63] Pearson T., Hornstra H., Hilsabeck R., Gates L., Olivas S., Birdsell D., Hall C., German S., Cook J., Seymour M. (2014). High prevalence and two dominant host-specific genotypes of *Coxiella burnetii* in U.S. milk. BMC Microbiol..

[64] Reichel R., Mearns R., Brunton L., Jones R., Horigan M., Vipond R., Vincent G., Evans S. (2012). Description of a *Coxiella burnetii* abortion outbreak in a dairy goat herd, and associated serology, PCR and genotyping results. Res. Vet. Sci..

[65] Kondo M., Dalai S.C., Venkatasubrahmanyam S., Eisenberg N., Robinson B.D., Westblade L.F., Marks K.M. (2019). Diagnosis and Genotyping of *Coxiella burnetii* Endocarditis in a Patient with Prosthetic Pulmonary Valve Replacement Using Next-Generation Sequencing of Plasma Microbial Cell-Free DNA. Open Forum Infect. Dis..

[66] Mioni M.D.S.R., Sidi-Boumedine K., Dalanezi F.M., Joaquim S.F., Denadai R., Teixeira W.S.R., Labruna M.B., Megid J. (2019). New Genotypes of *Coxiella burnetii* circulating in Brazil and Argentina. Pathogens.

[67] Astobiza I., Tilburg J., Piñero A., Hurtado A., García-Pérez A., Nabuurs-Franssen M., Klaassen C. (2012). Genotyping of *Coxiella burnetii* from domestic ruminants in northern Spain. BMC Vet. Res..

[68] Klaassen C.H.W., Nabuurs-Franssen M.H., Tilburg J.J.H.C., Hamans M.A.W.M., Horrevorts A.M. (2009). Multigenotype Q Fever Outbreak, the Netherlands. Emerg. Infect. Dis..

[69] Račić I., Špičić S., Galov A., Duvnjak S., Zdelar-Tuk M., Vujnović A., Habrun B., Cvetnić Ž. (2014). Identification of *Coxiella burnetii* genotypes in Croatia using multi-locus VNTR analysis. Vet. Microbiol..

[70] Ceglie L., Guerrini E., Rampazzo E., Barberio A., Tilburg J.J.H.C., Hagen F., Lucchese L., Zuliani F., Marangon S., Natale A. (2015). Molecular characterization by MLVA of *Coxiella burnetii* strains infecting dairy cows and goats of north-eastern Italy. Microbes Infect..

[71] Santos A.S., Tilburg J.J.H.C., Botelho A., Barahona M.J., Núncio M.S., Nabuurs-Franssen M.H., Klaassen C.H. (2012). Genotypic diversity of clinical *Coxiella burnetii* isolates from Portugal based on MST and MLVA typing. Int. J. Med. Microbiol..

[72] Sulyok K.M., Hornok S., Abichu G., Erdélyi K., Gyuranecz M. (2014). Identification of Novel *Coxiella burnetii* Genotypes from Ethiopian Ticks. PLoS ONE.

[73] Tilburg J.J.H.C., Roest H.-J.I.J., Buffet S., Nabuurs-Franssen M.H., Horrevorts A.M., Raoult D., Klaassen C.H.W. (2012). Epidemic Genotype of *Coxiella burnetii* among Goats, Sheep, and Humans in the Netherlands. Emerg. Infect. Dis..

[74] Joulié A., Sidi-Boumedine K., Bailly X., Gasqui P., Barry S., Jaffrelo L., Poncet C., Abrial D., Yang E., Leblond A. (2017). Molecular epidemiology of *Coxiella burnetii* in French livestock reveals the existence of three main genotype clusters and suggests species-specific associations as well as regional stability. Infect. Genet. Evol..

[75] Cumbassá A., Barahona M.J., Cunha M.V., Azórin B., Fonseca C., Rosalino L.M., Tilburg J., Hagen F., Santos A.S., Botelho A. (2015). *Coxiella burnetii* DNA detected in domestic ruminants and wildlife from Portugal. Vet. Microbiol..

[76] Boarbi S., Mori M., Rousset E., Sidi-Boumedine K., Van Esbroeck M., Fretin D. (2014). Prevalence and molecular typing of *Coxiella burnetii* in bulk tank milk in Belgian dairy goats, 2009–2013. Vet. Microbiol..

