# Supplementary figures and images for: Correlating Genotyping Data of Coxiella burnetii with Genomic Groups
Source: Pathogens. 2021 May 14;10(5):604. doi: 10.3390/pathogens10050604 (PMC8156542; doi:10.3390/pathogens10050604)

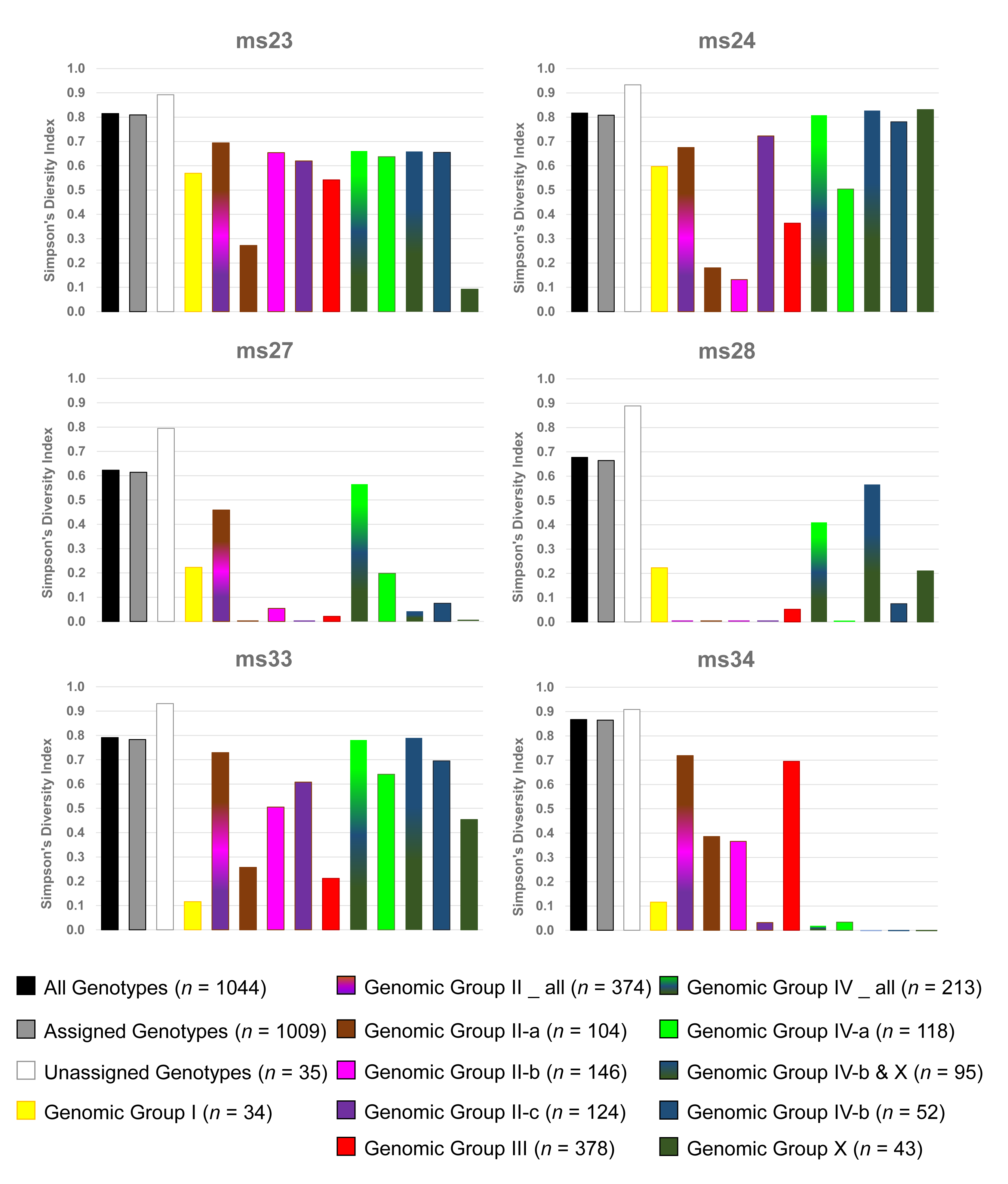

Supplement: Supplementary file 1 [file pathogens-10-00604-s001.zip › Fig_S1.tif]

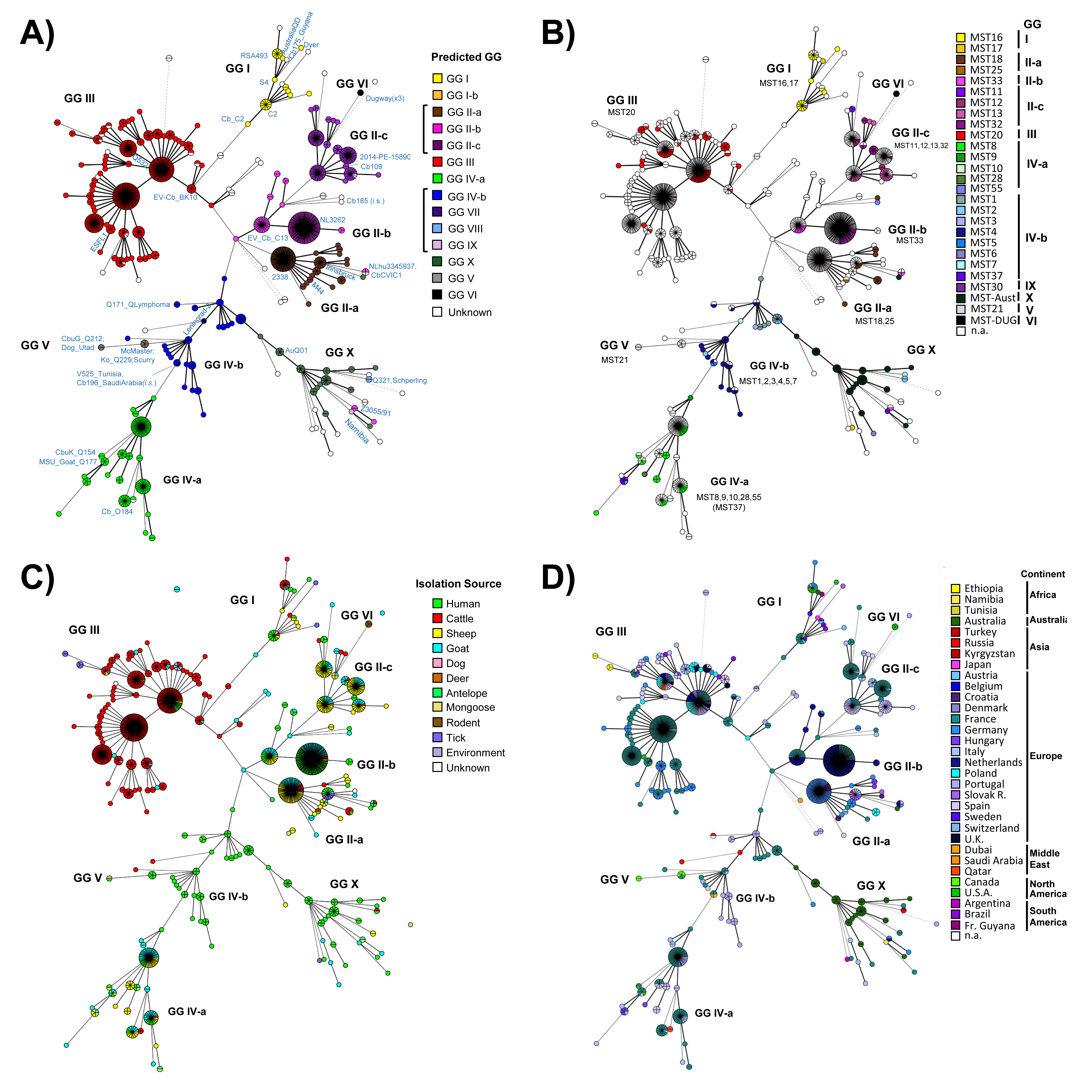

Supplement: Supplementary file 1 [file pathogens-10-00604-s001.zip › Fig_S2.tif]

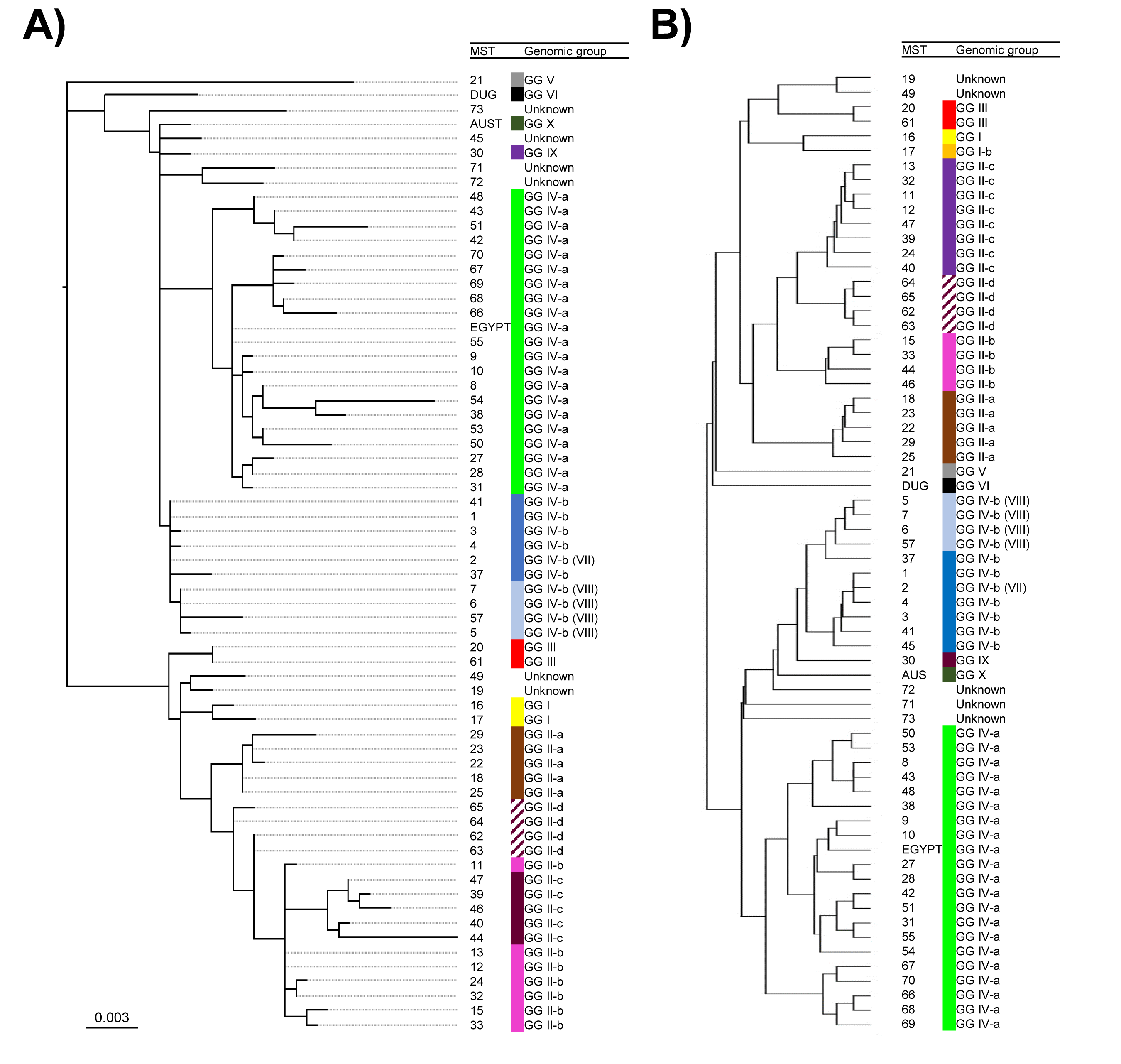

Supplement: Supplementary file 1 [file pathogens-10-00604-s001.zip › Fig_S3.tif]

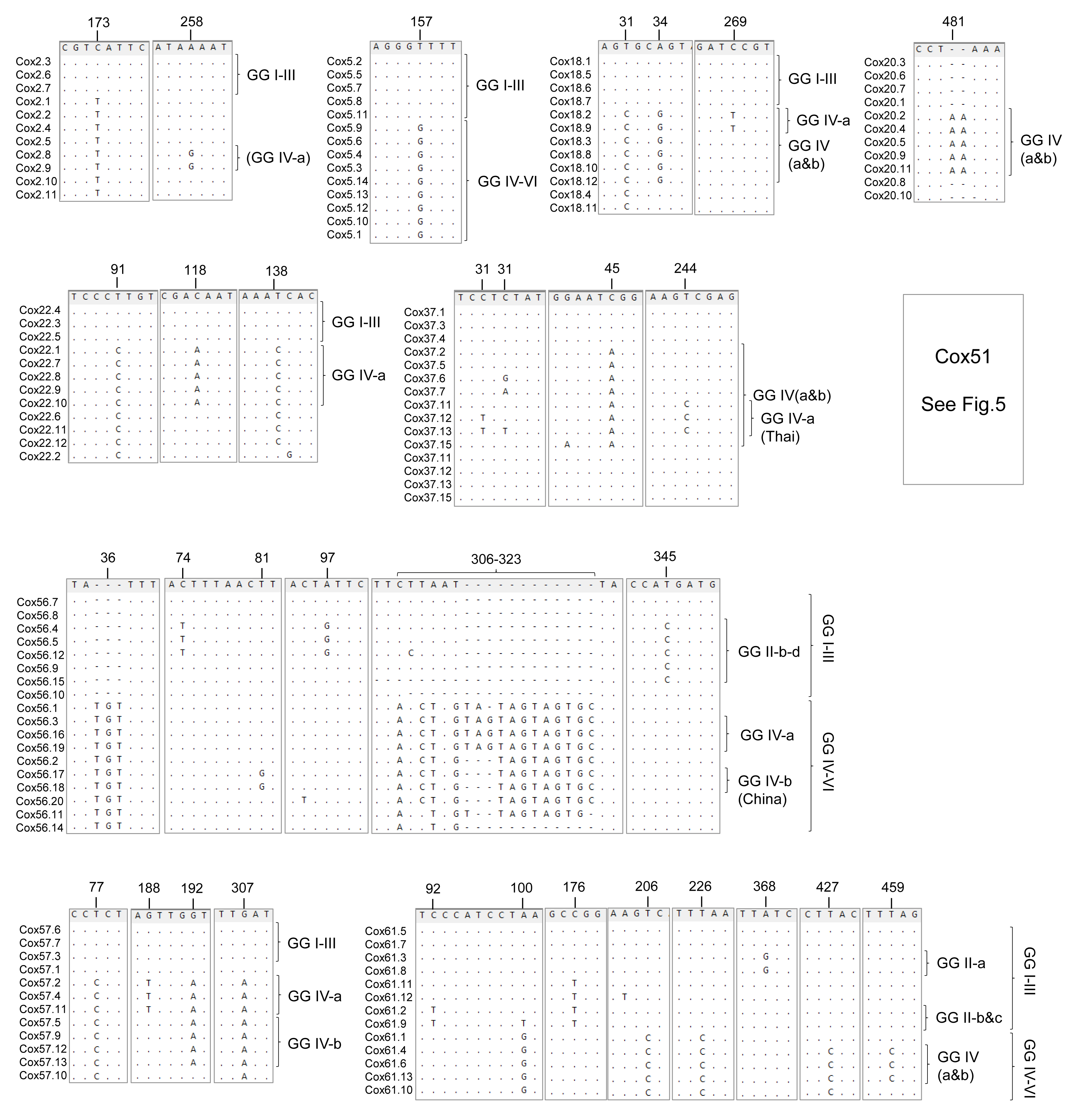

Supplement: Supplementary file 1 [file pathogens-10-00604-s001.zip › Fig_S4.tif]

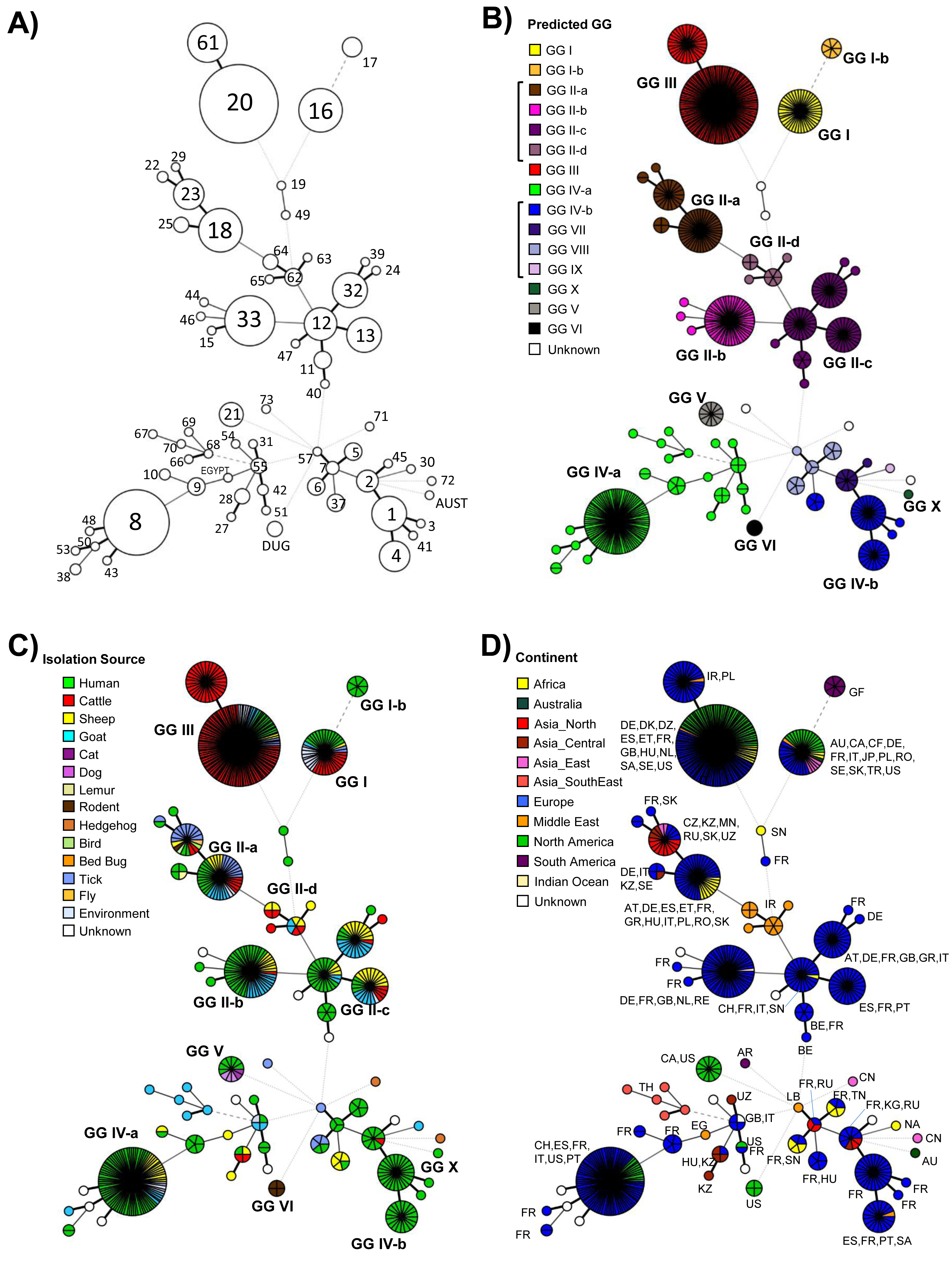

Supplement: Supplementary file 1 [file pathogens-10-00604-s001.zip › Fig_S5.tif]

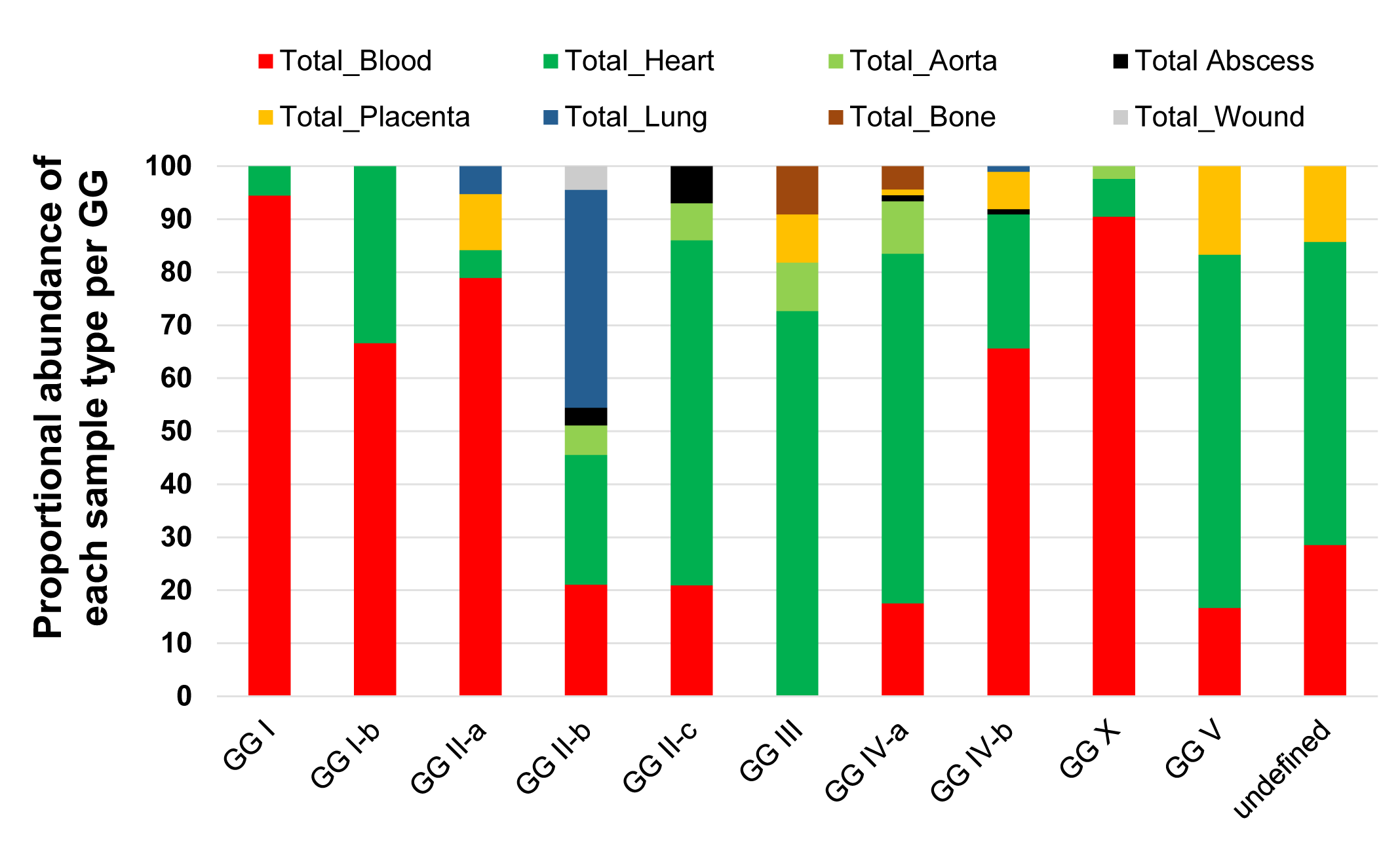

Supplement: Supplementary file 1 [file pathogens-10-00604-s001.zip › Fig_S6.tif]

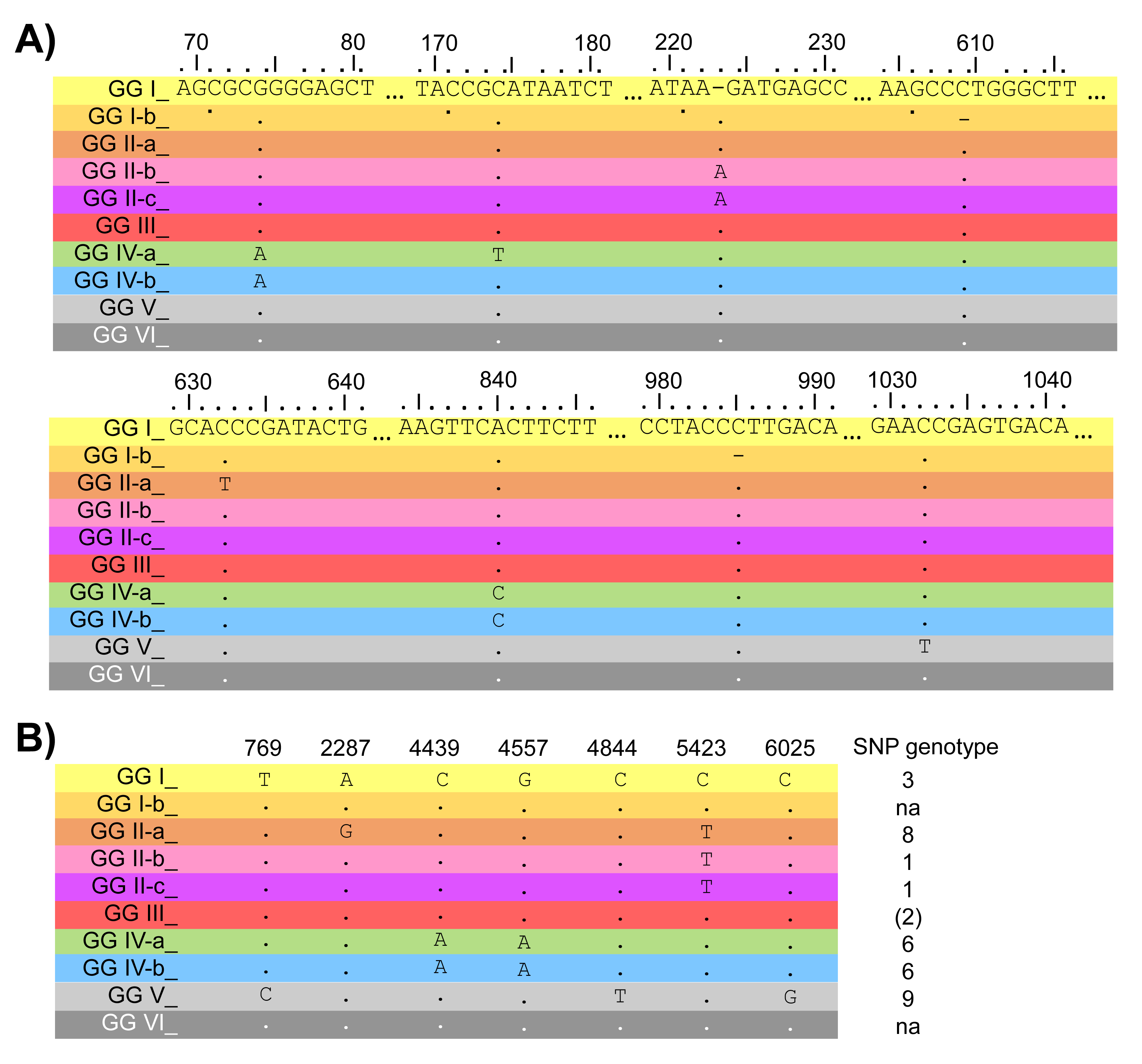

Supplement: Supplementary file 1 [file pathogens-10-00604-s001.zip › Fig_S7.tif]
